# The A's, G's, C's, and T's of health disparities

**DOI:** 10.1186/1755-8794-2-29

**Published:** 2009-05-22

**Authors:** Edward Ramos, Charles Rotimi

**Affiliations:** 1 Center for Research on Genomics and Global Health, National Human Genome Research Institute, National Institutes of Health, 12 South Drive, Bethesda, MD 20892-5635, USA

## Abstract

In order to eliminate health disparities in the United States, more efforts are needed to address the breadth of social issues directly contributing to the healthy divide observed across racial and ethnic groups. Socioeconomic status, education, and the environment are intimately linked to health outcomes. However, with the tremendous advances in technology and increased investigation into human genetic variation, genomics is poised to play a valuable role in bolstering efforts to find new treatments and preventions for chronic conditions and diseases that disparately affect certain ethnic groups. Promising studies focused on understanding the genetic underpinnings of diseases such as prostate cancer or beta-blocker treatments for heart failure are illustrative of the positive contribution that genomics can have on improving minority health.

## Background

**Figure 1:**
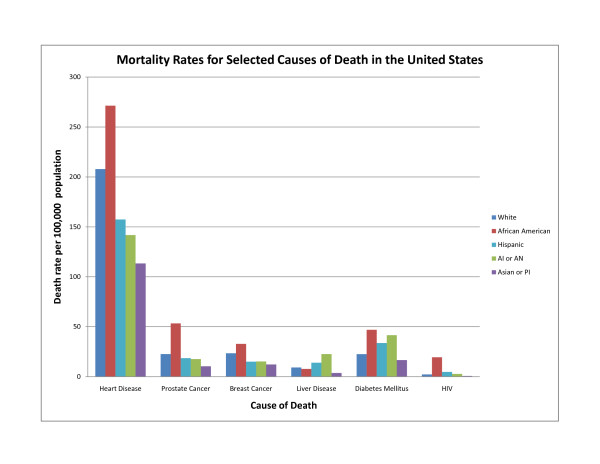
**Death rates of selected ethnicities for six causes of death in the United States**. Rates are per 100,000 population and age-adjusted to the 2000 census. AI = American Indian, AN = Alaska Native, PI = Pacific Islander. Source: Health, United States, 2007.

Disparities or inequities in health refer to socio-demographic group differences in the distribution of disease, health outcomes, or access to health care. In the United States, there is overwhelming evidence for the existence of disparities in health when ethnic minority groups (as defined by the Office of Management and Budget [1] but referred to here as ethnicities [2]) are compared to their white counterparts[3] (Figure 1). A number of factors play a significant role in varying health outcomes, which include, but are not limited to, socio-political structure, discrimination, cultural practices (e.g., diet), socioeconomic status, exposure to harmful toxins in the environment, and access to health care.

The battle against eliminating ethnic health disparities in the United States begins, and perhaps ends, at the social level. Therefore, one may question the relevance and utility of a genetics lens as a means to view these disparities. By focusing on new insights on the global pattern of human genetic variation (HGV), made possible by the successful completion of the International HapMap Project [4] and ongoing sequencing efforts of individual genomes, this article will provide important illustrations of how genomics may inform our understanding of population differences in disease distribution and variable drug response.

## Human genetic variation: Understanding our similarities and differences

**Figure 2:**
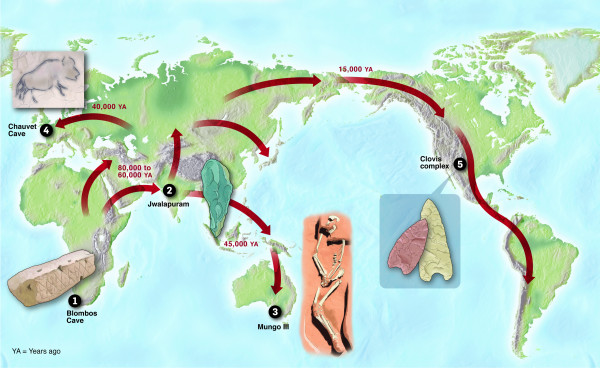
**Human migration pattern from Africa to Asia, Australia, Europe, and North and South America**. Selected artifacts found around the world (1–5) are examples of evidence supporting the out-of-Africa theory. Reprinted with permission from 5W Infographics.

In order to appreciate the sources of HGV, it is important to understand the common and unique histories of human populations. Fossil evidence dates the rise of *Homo sapiens *at approximately 200,000 years ago [5-7]. Although the migration known as the out-of-Africa theory[8,9] took place approximately 80,000 years ago (Figure 2), many experts would consider all *Homo sapiens *to be Africans as recent as 36,000 years ago (modern humans had already reached as far as Europe and Australia by this point) based on evidence from cranial analysis and dating of a Late Pleistocene human skull [10]. As modern humans spread throughout the world, the frequency of genetic variations varied from region to region as a result of random chance, natural selection, and other genetic mechanisms[11]. In short, our diversity, genetic or otherwise, is not an illusion. It is intimately associated with our journey within and out of Africa and it is the foundation of our uniqueness: from physical characteristics to disease susceptibility or resistance.

Genetic variations can occur at different frequencies in different populations, especially when those populations are widely separated and unlikely to exchange much genetic material through mating. Interestingly, the most recently arising variants have not had enough time to spread widely beyond the population and geographic region in which they originated. For this reason, there must remain a consistent link between HGV and the historic and cultural experiences of human populations as we look to understand differential disease distribution and variable drug response.

## Human genetic variation and disease

From a biological perspective, the genetic underpinnings of many diseases remain to be described in complete detail or at all. It is rare to stumble across scenarios in which mistakes in the genetic alphabet are contained within a single gene and lead to a specific disease. Sickle cell disease, cystic fibrosis, fragile-X syndrome, Huntington's disease, and other single-gene disorders affect less than two percent of the general population. These diseases often have a simple and predictable inheritance pattern and their manifestation is largely independent of social determinants. Unfortunately, it is now clear that the formula for identifying the underlying cause for common diseases (e.g., diabetes, heart disease, and cancer) is more complex than the examples mentioned above. However, the common theme remains that these diseases are genetic in nature, triggered or influenced in varying degrees by non-genetic forces such as diet, stress, or exposures to harmful agents in the environment.

There have been a number of scientific advancements made in the past decade that allow for our genetic code to be analyzed at a fantastic rate, which yields faster and more accurate identification of genetic mutations [12]. Information garnered from such analyses has tremendous potential to lend insight into the genetic mechanisms of disease and disease susceptibility. For example, the agnostic search of thousands of human genomes (e.g., genome-wide association studies – GWAS) is rapidly shedding light on subsets of genetic variants that are associated with an increase risk of particular conditions[13,14]. These findings can also provide clarification on the pervasiveness of different genetic variants across or within ethnic groups, which can potentially influence the way we treat and diagnose disease. We present working examples of the potential impact of genomics on variable drug response and disparities in health at the individual and group levels.

## Group and individual identity in the genomic era: Lessons from variable response to drugs

The Centers for Disease Control and Prevention recognizes cardiovascular disease as a condition that disproportionately affects ethnic minorities. The 2007 National Center for Health Statistics report shows that African Americans have the highest rate of hypertension when compared to Hispanic or White populations in the United States [3]. Hypertension, in turn, has been identified as a significant risk factor for stroke and myocardial infarction, which can often lead to cardiac failure and ischemia. The statistics are sobering: almost 300,000 Americans die from heart failure every year [3] and one out of four heart failure patients dies within a year of diagnosis and one out of two within five years [15,16]. Socioeconomic indicators such as income and educational levels are powerful predictors but fall short of completely explaining the incidence of this disease in different populations [17].

From the clinical perspective, the standard care for treating heart failure is administration of beta-adrenergic receptor (betaAR) blocking agents, or beta-blockers, that act by suppressing the action of the hormone adrenaline. However, over the past several years the medical community found it difficult to arrive at a consensus on the efficacy of beta-blockers in African Americans. Confounding reports between 1999 and 2002 added to the confusion of whether race played a role or not in how individuals responded to beta-blockers and the medical community was left to rely on anecdotal or inconclusive evidence [18-22].

Last year, Stephen Liggett and colleagues provided a genetic explanation for the perceived disparate health outcomes observed among African Americans following the administration of beta-blockers [23]. The authors published a well-designed study that revealed a nonsynonymous polymorphism in the G protein-coupled receptor kinase 5 (i.e., leucine is substituted for glutamine at position 41) which they showed confers a "natural genetic beta-blockade." Individuals that carry this variant (GRK5-Leu41) have an increased survival rate against cardiac failure and ischemia when compared to those without the protective mutation when no betaAR antagonists are administered. Moreover, the difference in time to cardiac transplant or mortality was not significant when comparing GRK5-Leu41 individuals to patients who received beta-blockers but did not carry the protective variant.

Support for Liggett and colleagues' conclusions stems from the comprehensive nature of their approach. The authors employed sequencing and genotyping, pharmacogenomics, transgenic mouse models, rigorous statistical analysis, and robust human study protocols to describe and ultimately confirm the genetic rationale for the confusing efficacy of beta-blockers in African Americans. However, the take-home message goes beyond genetic sleuthing. The findings provide meaningful evidence to support the need for a paradigm shift (i.e., genetic profiling in clinical trials) as treatments move closer to individualized medicine. Group labels such as African Americans fall short as a reliable predictor for how a member of this group will respond to medications such as beta-blockers. In essence, having the protective variant is not a defining characteristic of African Americans since only a proportion, approximately 40%, carry it. African Americans who do not carry this variant derive significant benefits from beta-blockers in the treatment of heart failure. Therefore, the end result of classifying and subsequently treating African Americans as one uniform group is an unclear and misrepresented interpretation of beta-blocker efficacy, which potentially puts a majority of African Americans at risk of not receiving the proper treatment. The confusion on whether or not beta-blockers are effective in African Americans highlights a prevailing notion, among the medical and scientific communities alike, that the group "African American" is genetically and culturally homogenous.

The problem of using group data as a proxy for individuals in the context of drug response is not limited to historically-labeled admixed populations such as African Americans. This point was demonstrated clearly in a recent commentary by Ng and colleagues [24]. The examination of the complete sequence of the personal genomes of two Caucasian men (J. Craig Venter [25] and James Watson [26]) revealed that the group label "White" or "Caucasian" was inadequate in predicting their metabolic status with respect to key drug-metabolizing genes. This observation underscores the need to know individual genetic variants instead of relying on a patient's appearance or self-identified ethnicity.

## Genomics and health disparities: Lessons from the genetics of prostate cancer

Prostate cancer provides another striking example of ethnic minorities disproportionately affected by disease. In the United States, an estimated 186,000 new cases for prostate cancer tops the 2008 list of incident cancer cases in men [27]. Moreover, African-American men unequally share the burden of this disease, which is illustrated both in incidence and in mortality (approximately 1.6- and 2.4-fold higher than European Americans, respectively) [27]. Numerous reports have indicated that disparities exist in treatment and access to adequate health care, which ultimately contribute to disparities in mortality rates [28]; of note, breast cancer mortality is higher in African-American women despite a greater incidence in European American women [29]. It is therefore critically important to approach the problem of group differences in complex disease susceptibility in a comprehensive manner.

In the case of prostate cancer, genetics provides compelling, though preliminary data on the potential role of molecular factors in explaining the significant unbalanced incidence of prostate cancer in African-American and potentially other men from populations of the African diaspora. Several independent studies have identified and replicated genomic regions that contain risk variants for prostate cancer in multiple human populations [30-33]. A locus on chromosome 8, specifically 8q24, has reproducibly been associated with prostate cancer in men from several ancestral backgrounds including Europeans, Africans, Latinos, and Japanese [34]. The impact of these susceptibility variants on prostate cancer varies significantly with ancestry ranging from population attributable risks (PAR) of 8% to 68% [33]; the PAR is defined as the number (or proportion) of cases that would not occur if the risk factor were eliminated. Interestingly, the PAR for all identified variants in the 8q24 region is 32% in European Americans and 68% in African Americans. If functional studies confirm these alleles as "true" susceptibility variants, the observed differences in allele frequencies and PAR between Europeans and African Americans may explain a significant proportion of the disparity in prostate cancer incidence between the two groups. It is important to point out that none of the genetic variants identified in the 8q24 region lie within known genes or alter the coding sequence of an encoded protein [34].

The examples described in this section are not of rare or obscure diseases. The impact of these diseases on our general population is severe and the disproportionate impact on subgroups within the population is, by definition, unequal. Genetics must continue to play a significant role alongside social and behavioral research and social service programs and initiatives.

## Genomics, group identity and health disparities

Genomics research has much to offer in the global effort to understand disease susceptibility and resistance at the individual and population levels [35]. However, genetics and, by association, genomics are certainly not immune to producing a certain level of controversy and confusion with respect to the communication and interpretation of data. In the fervor of discovery, researchers at times forget or, perhaps, they do not feel it is their obligation, to take a step back and put their work in an appropriate context. Finding a novel mechanism associated with a biochemical pathway can be equally important as linking genetic variants to disease susceptibility; however, the parallels may end when viewed from a social perspective.

Microarrays spotted with hundreds of thousands of human single nucleotide polymorphisms and high-throughput sequencing and genotyping efforts have all contributed to the vast amounts of data that are literally streaming into research laboratories for analysis [36]. Population-genetics studies have turned from a science du jour to a necessary and integral part of how we identify disease susceptibility among populations with ancestry from different parts of the world. The trend is in part dictated by technology, which only seems to be getting faster and cheaper. The pace of progress shows no sign of slowing down and the stream of information will only get broader. However, we cannot shy away from the potential pitfalls that inherently lurk when assumptions and conjecture are hazily combined with empirical data especially since these pitfalls are potentially larger and deeper when comparisons across ethnic groups are involved.

Lohmueller *et al. *recently published a population-based study that investigated the abundance of deleterious mutations found among European and African populations [37]. Their findings led them to conclude that European Americans have acquired genetic variations considered to be harmful in greater number when compared to African Americans. The classification of "possibly damaging" and "probably damaging" prompted disagreement among other researchers not involved in the study. While the discrepancy in interpretation may be nuanced, the potential impact of misinterpreting the broader statements made on these different populations appears far greater when put in a social setting. We do not expect the authors to be at fault for nonsensical arguments based on poor generalizations. However, the larger point is that health disparities are so heavily rooted in social structure that the distribution and dissemination of genomic data must acknowledge the broader context in order to comprehensively educate both the scientific community and the public.

## Conclusion

Health disparities are a global phenomena by no means limited to the United States. The social determinants that feed these health inequalities and inequities [39,40] are undeniable. Geoffrey Rose, a revered epidemiologist whose insight furthered our current understanding of public health, is often cited for his exclamation to search for the "causes of the causes". Many have justly viewed this statement as a challenge to change public policy and a call for an equal and fair distribution of societal resources. While a sigh of relief is pending, we can find solace in knowing that much of what continues to widen the healthy divide – poor education, substandard living and working conditions, limited access to affordable healthcare – is reversible. However, the origin of health differences is indeed complex and biology cannot be left out of the discussion if we are to understand why some individuals get certain diseases and others do not or why some respond to treatment differently. To that end, the contributions of genetics and genomics should not be viewed as a distraction but, rather, as a positive addition to the collective efforts in eliminating health disparities globally.

In the search to understand biology's role in health differences, the scientific community, particularly genetic and genomic investigators, should make considerable effort to interpret their data regarding group differences within the context of the historic experiences of these groups. It is also important to recognize the challenge posed by the use of "race" in biomedical research. The use of social group labels such as African American, Hispanics, and Asians are likely to be insufficient to get us to where we need to be as we strive towards individualized medicine. For this reason, the emphasis falls squarely on the study of human genetic variation. As mentioned previously [2], understanding the detailed structure of human genetic variation may help to deconstruct imprecise group definitions currently applied in biomedical research and avoid unintended consequences of generalizing biological characteristics across these groups (e.g., beta-blocker efficacy).

If we use genomic information correctly, we will simultaneously describe our similarities and differences without reaffirming old prejudices. More importantly, the careful unbiased study and interpretation of the human story coded in our DNA will enable us to appreciate the fact that individuals cannot be treated as a representative for all those who physically resemble them or who share some of their ancestry. The human genome is a mosaic of our experiences, past and present.

## Abbreviations

betaAR: beta-adrenergic receptor; GWAS: Genome-wide association studies; HGA: Human genetic variation; PAR: population attributable risks

## Competing interests

The authors declare that they have no competing interests.

## Pre-publication history

The pre-publication history for this paper can be accessed here:


